# Combined Inhibition of IGF-1R/IR and Src Family Kinases Enhances Antitumor Effects in Prostate Cancer by Decreasing Activated Survival Pathways

**DOI:** 10.1371/journal.pone.0051189

**Published:** 2012-12-26

**Authors:** Farshid Dayyani, Nila U. Parikh, Andreas S. Varkaris, Jian H. Song, Shhyam Moorthy, Tanushree Chatterji, Sankar N. Maity, Adam R. Wolfe, Joan M. Carboni, Marco M. Gottardis, Christopher J. Logothetis, Gary E. Gallick

**Affiliations:** 1 Department of Genitourinary Medical Oncology and David H. Koch Center for Applied Research of Genitourinary Cancers, The University of Texas MD Anderson Cancer Center, Houston, Texas, United States of America; 2 Program in Cancer Metastasis, The University of Texas Graduate School of Biomedical Sciences at Houston, Houston, Texas, United States of America; 3 Department of Pharmaceutical Candidate Organization, Bristol-Myers Squibb Company, Princeton, New Jersey, United States of America; 4 The University of Texas Graduate School of Biomedical Sciences at Houston, Houston, Texas, United States of America; University of Nebraska Medical Center, United States of America

## Abstract

**Background:**

Treatment of metastatic prostate cancer (PCa) with single agents has shown only modest efficacy. We hypothesized dual inhibition of different pathways in PCa results in improved tumor inhibition. The Src family kinases (SFK) and insulin-like growth factor-1 (IGF-1) signaling axes are aberrantly activated in both primary PCa and bone metastases and regulate distinct and overlapping functions in PCa progression. We examined the antitumor effects of combined inhibition of these pathways.

**Materials and Methods:**

Src andIGF-1 receptor (IGF-1R) inhibition was achieved *in vitro* by short hairpin (sh)RNA and *in vitro* and *in vivo* by small molecule inhibitors (dasatinib and BMS-754807, against SFK and IGF-1R/Insulin Receptor(IR), respectively).

**Results:**

*In vitro*, inhibition of IGF-1 signaling affected cell survival and proliferation. SFK blockade alone had modest effects on proliferation, but significantly enhanced the IGF-1R blockade. These findings correlated with a robust inhibition of IGF-1-induced Akt1 phophorylation by dasatinib, whereas Akt2 phosphorylation was SFK independent and only inhibited by BMS-754807. Thus, complete inhibition of both Akt genes, not seen by either drug alone, is likely a major mechanism for the decreased survival of PCa cells. Furthermore, dasatinib and BMS-754807 inhibited *in vivo* growth of the primary human xenograft MDA PCa 133, with corresponding inhibition of Akt in tumors. Also, both orthotopic and intratibial tumor growth of PC-3 cells were more potently inhibited by dual SFK and IGF-1R/IR blockade compared to either pathway alone, with a corresponding decrease in bone turnover markers.

**Conclusions:**

Dual IGF-1R/IR and SFK inhibition may be a rational therapeutic approach in PCa by blocking both independent and complementary processes critical to tumor growth.

## Introduction

Metastatic prostate cancer (PCa) accounts for an estimated 28,000 deaths in 2012 [1]. In advanced, castrate-resistant metastatic PCa (CRPC), few treatment options exist. There are only 3 FDA-approved chemotherapeutic agents for CRPC: docetaxel and cabazitaxel [2], [3], both of which show only a modest survival advantage for men with CRPC, and more recently, the CYP17 inhibitor abiraterone acetate, approved for use after docetaxel failure (ClinicalTrials.gov Identifier: NCT00638690), improving overall survival by 3.9 months [4]. For minimally symptomatic metastatic CRPC, a randomized phase 3 trial showed survival advantage with the vaccine Sipuleucel-T [5].Thus, while promising agents are improving survival of PCa patients, additional therapeutic agents are clearly needed for advanced stage disease [6].

Recent advances in the understanding of the signaling pathways governing growth of PCa cells in the bone have led to numerous clinical trials with targeted therapies. One of the most promising targets in PCa are the Src family kinases (SFKs), a family of non-receptor protein tyrosine kinases, the expression and specific activities of which are increased in multiple types of human tumors [7]. Preclinical data in PCa show that inhibition of SFKs decreases proliferation and, more strongly, invasion and migration [8], [9]. In addition, Src activity is important in the microenvironment, affecting osteoclast function. Thus, Src inhibitors block, in part, tumor/microenvironment interactions that lead to the “vicious cycle” of bone metastasis [10]. In studies using *in vivo* orthotopic mouse experiments, inhibition of SFKs decreased both prostate tumor growth and development of lymph node metastases [11]. Based in part on these findings and some promising results from a phase 1/2 trial [12], dasatinib, a small molecule multi-kinase inhibitor with selectivity forSFK/Abl [8], has been tested in combination with docetaxel in a randomized phase 3 trial for patients with CRPC (ClinicalTrials.gov ID: NCT00744497).

Analyses of the phase 1/2 trial indicate SFK inhibition plus docetaxel did not produce meaningful clinical responses in a majority of patients, but were extremely promising for a subset of patients [13]. Thus, identifying additional signaling pathways that contribute to the metastatic growth of PCa and may augment the effects of Src inhibition is likely to yield promising combinations of inhibitors that will be more efficacious for treatment of PCa.

One such pathway deregulated in PCa is mediated by the insulin-like growth factor-1 receptor (IGF-1R). IGF-1R is implicated in proliferation and survival of many tumor types [14] and is overexpressed in PCa [15]. IGF-1R signaling has been linked to PCa risk, and one of its ligands, IGF-2, is also overexpressed in PCa bone metastases [16], where it promotes proliferation and survival of PCa cells [17], [18], [19], [20], [21]. Studies with monoclonal antibodies to IGF-1R have implicated this receptor as important in PCa progression in androgen-sensitive as well as -resistant tumor models [22]. Although IGF-1R signaling is mediated in part by the Src pathway, other downstream signaling pathways of IGF-1R are Src independent and unaffected by Src inhibition [14]; these additional pathways have been shown recently to play an important role in PCa cell survival [23]. Activating Src and IGF-1R also activates Akt, a key effector of the PI3K/Akt/mTOR pathway, which is aberrantly activated in the majority of malignancies, promoting cell growth, proliferation, and survival [24], [25].However, whether these inhibitors are equally effective in inhibiting Akt1, 2, and 3 functions is not known, and if combining them produced better results in inhibiting this survival pathway was a goal of this work. Here, we examined the individual and combined effects of dasatinib, an SFK inhibitor, and BMS-754807, a potent and reversible small molecule inhibitor of IGF-1R [26] and the insulin receptor (IR) with activity against various solid tumors *in vitro* as well as in multiple xenograft models [26]. As inhibitors have off-target effects, molecular knockdown of these pathways was also employed. We demonstrate that inhibition of these pathways yields complementary biologic effects *in vitro* and *in vivo* and that, in the presence of IGF-1, inhibition of both pathways is required to potently inhibit Akt phosphorylation and downstream mediators of cancer cell survival.

## Materials and Methods

### Cell cultures

PC-3 and LNCaP cells were obtained from the American Type Culture Collection. They represent both adeno- and small-cell variants of PCa [27]. PC3-MM2 [28] and PC3-LG cells [29] were kindly provided by Dr. Isaiah Fidler (The University of Texas MD Anderson Cancer Center). PC-3 wild-type cells and stable transfectants were maintained in DMEM F-12 medium (Hyclone) supplemented with 10% fetal bovine serum (FBS;Hyclone) and 1% penicillin/streptomycin (Gibco). LNCaP wild-type cells and stable transfectants were maintained in RPMI 1640 medium (Hyclone) supplemented with 10% FBS and 1% penicillin/streptomycin. BMS-754807 was provided by Bristol-Myers Squibb, and dasatinib was kindly provided by Dr. John C. Araujo (MD Anderson). Cell authentication and mycoplasma screening was performed every six months according to institutional guidelines.

### Stable transfectants

Ready-to-transfect short hairpin (sh) RNA–GFP–puromycin constructs against human IGF1-R (#SR302344) and Src(#SR304574) were purchased from OriGene Technologies. A universal scrambled negative control shRNA (#SR30004) was provided by the manufacturer. Cells were transfected using Fugene 6 reagent (Roche) according to the manufacturer's instructions. Stable clones were selected with puromycin. Target knockdown was verified 3–4 weeks after transfection by western blots, as described below.

### Cell proliferation assay

Cells (3×10^4^per well) were cultured in 6-well dishes for up to 96 hours with and without dasatinib (100 nM) or BMS-754807 (0.5–5 µM). For cell counting at various time points, cells were washed once with PBS (Gibco), incubated with TrypLE dissociation reagent (Gibco), and counted using an automated cell viability analyzer (Vi-Cell XR;Beckman Coulter). All experiments were performed in triplicate.

### Annexin V staining

Staining of apoptotic cells was performed using an annexin V-PE detection kit (BD Pharmingen) according to the manufacturer's instructions. Briefly, cells were cultured for 48 hours, followed by staining with annexin V-PE. Cells were analyzed on a BD FACS Canto flow cytometer, using the FACS Diva software (BD Biosciences).

### Cell cycle

Cells were cultured for 24 hours, harvested, and washed once in PBS, then resuspended in ice-cold 70% ethanol and kept at −20°C for 1 hour. Cells were then washed again in PBS and treated for 45 minutes with DNAse-free RNAse (Invitrogen) at 37°C. Propidium iodide was added at a final concentration of 1 µg/mL, and the cells were incubated for 20 minutes at room temperature. Analysis was done on a BD FACS Canto flow cytometer, using the FACS Diva software.

### Animals

Swiss nu-nu/Ncr nude mice were purchased from the animal production area of the Department of Experimental Radiation Oncology at MD Anderson. All mice were housed and maintained under specific pathogen–free conditions according to MD Anderson's Institutional Animal Care and Use Committee guidelines. The MD Anderson Institutional Animal Care and Use Committee (IACUC) reviewed this study and specifically approved it.

### Xenograft tumors

Subcutaneous grafts of MDA PCa 133 (a xenograft from a castrate-resistant patient derived from a bone metastasis that expresses AR and PSA and grows only in mice and not in cell culture) were generated as described previously [30]. In brief, fragments of tumors were implanted subcutaneously into severe combined immunodeficient (SCID) mice at a size less than 1 mm^3^. The tumors were allowed to grow until they reached the size of 3 mm^3^, and then the mice (n = 4 for each group) were treated daily with dasatinib and BMS-754807, alone and in combination, for 26 days. Tumors were measured twice weekly, and the volume was calculated using the formula *V = W^2^× L/2*. On day 26, the mice were euthanized, the tumors were harvested, and protein lysates were prepared as stated below for western blots.

### Orthotopic prostatic/intratibial injections

Intraprostatic injection of luciferase-expressing PC3-LG cells was done as previously published by our group [31]. Briefly, 40 Swiss nu-nu/Ncr nude mice (8–12 weeks old) were anesthetized with 2% isoflurane in an isoflurane-oxygen chamber and then placed in a supine position. A midline incision was made on the lower abdomen, and the prostate was exteriorized as described by Park et al. [32]. Fifty microliters of HBSS containing PC3-LG (total, 5×10^5^ cells) was orthotopically injected as described previously [31] into a lateral lobe of the prostate. The wound was closed with metal surgical clips. Ten days after the xenograft injection, tumor engraftment was determined by *in vivo* bioluminescence imaging (IVISTM 100 system;Xenogen Co.) with the mice under 2% isoflurane inhalation anesthesia. Thirty-two mice showing luciferase activity close to the median value were selected and randomized by using an Excel® (Microsoft) program into 4 groups(n = 8 for each group): control vehicle, BMS-754807 alone (12.5 mg/kg body weight/day), dasatinib alone (12.5 mg/kg body weight/day), and a combination of BMS-754807 (6.25 mg/kg body weight/day) and dasatinib (12.5 mg/kg body weight/day). These concentrations were deliberately chosen at levels in which single-agent activity was not expected. All drugs were administrated by oral gavage 6 days/week. The mice were euthanized 4 weeks after injection of cells. Primary tumors in the prostate were excised and weighed.

Intratibial injection of PC3-MM2 cells was performed as previously described [28]; briefly, nude mice were anesthetized as above, and 2.5×10^5^ cells were injected into the tibia as described [28]. Twelve days after injection, the mice were treated with saline (n = 10) dasatinib (n = 9), BMS-754807 (n = 9), or both(n = 8) at the dosages described above. Four weeks after treatment initiation, the mice were euthanized. X-rays were taken of all mice, and from 4 mice in each group, the tibiae were harvested and prepared, and computed tomography was performed as described previously [32], [33].

Bone destruction after intratibial injection of PC3-MM2 cells in nude mice was graded based on the following scores: 0 = Minimal changes; 1 = Lytic bone lesions, cortex intact; 2 = Cortex destruction, no significant soft tissue swelling; 3 = Cortex destruction, significant soft tissue involvement.

### Immunoblotting

Immunoblotting was performed as described previously [34]. Briefly, cells were lysed and clarified, and the proteins were separated via 8% SDS-PAGE, followed by transfer onto polyvinylidenedifluoride membranes (Millipore). The membranes were incubated with antibodies against Src, Yes, Fyn, Lyn, IGF-1R, Akt, Erk1/2, and S6 (all purchased from Cell Signaling) and LC3 (Sigma), followed by horseradish peroxidase–conjugated secondary antibodies (Bio-Rad).

### Immunoprecipitation

To determine phosphorylation of Akt1 and 2 specifically, PC-3 cells were serum starved up to 48 hours and then incubated for 2 hours with dasatinib (100 nM), BMS-754807 (5 µM), or both, followed by 15-minute stimulation with 50 ng/mL rhIGF-1 (R&D Systems). The cells were then harvested, and 500 µg of protein was used for immunoprecipitation, as described previously [35]. The samples were incubated with specific antibodies against Akt1 or Akt2 (Cell Signaling) overnight at 4°C. Following immunoprecipitation, 50 µL of protein A agarose beads were added, and the samples were incubated again for 2 hours at 4°C. The samples were then centrifuged at 5000 rpm for 2 minutes. The supernatant was discarded, and each sample was washed with 500 µL of immune complex kinase assay wash buffer and centrifuged 3 times. Then, 30 µL of SDS dye was added to the precipitated proteins, and the samples were run for western blots and incubated with phospho-Akt (Cell Signaling) following the protocol described above.

### Histological studies

H&E and TUNEL staining were performed on MDA PCa 133 xenograft tumors after explantation as described previously [36], [37]. In each group, 5 different high-power fields (HPF) were examined, the number of TUNEL-positive cells was counted, and the average number of TUNEL-positive cells/HPF was calculated. Hoechst stain was used for staining cell nuclei.

### ELISA

Quantitative determination of murine alkaline phosphatase and N-telopeptide in mouse serum was performed using the respective ELISA kits (TSZ ELISA) according to the manufacturer's instructions. All standard curves had an R2 of ≥0.990. Positive controls for each assay were provided by the manufacturer.

### Statistics

For statistical comparisons of continuous parameters, the Student's *t*-test was used, and for discrete values, the chi-square test was used; α<0.05 was considered statistically significant.

## Results

### IGF-1R inhibition affects cell proliferation and apoptosis

**Figure 1:**
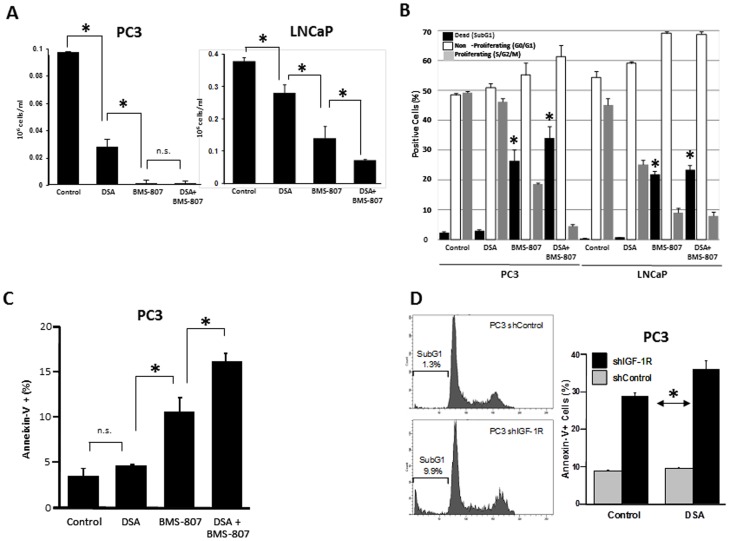
Dual inhibition of IR/IGF-1R and SFK decreases tumor growth *in vitro* more effectively than single pathway inhibition does. (A) PC-3 or LNCaP cells were cultured for 96 hours with and without dasatinib (DSA; 100 nM) and BMS-754807 (BMS-807; 2 µM for PC-3 and 0.5 µM for LNCaP cells), alone and in combination, and cell numbers were determined as described in the methods. (B) PC-3 and LNCaP cells were incubated for 24 hours with and without DSA (100 nM) and BMS-754807 (5 µM for PC-3 and 1 µM for LNCaP cells), alone and in combination, and cell cycle staining after 24 hours was performed using propidium iodide. (C) PC-3 cells were incubated for 48 hours with and without DSA (100 nM) and BMS-754807 (2 µM), alone and in combination, and the percentage of apoptotic cells was determined by flow cytometric analysis of annexin-V staining. (D)Left: cell cycle staining with propidium iodide in PC-3–shIGF-1R cells. Right: PC-3–shIGF-1R and control cells were incubated for 48 hours with and without DSA (100 nM), and the percentage of apoptotic cells was determined by flow cytometric analysis of annexin-V staining. All experiments were performed in triplicate. *P<0.05; N.S. not significant.

We tested our rationale for combined inhibition of the Src and IGF-1R pathways by determining the effects on growth and apoptosis then signal transduction, followed by effects on tumor growth in several relevant animal models. Cells were grown in 10% FBS, sufficient to lead to activated IGF-1R. Under these conditions, inhibition of Src and IGF-1R by the combination of dasatinib and BMS-754807 decreased cell proliferation in both PC-3 and LNCaP cells, as shown in Fig. 1A. First, we confirmed expression of the SFKs Src, Yes, Fyn, and Lyn in all PCa cells examined (Fig. S1A). To determine whether the inhibitors primarily affected Src and IGF-1R, stable shRNA-mediated knockdowns were made for *SRC* and *IGF-1R*, respectively. A >90% knockdown of the respective signaling enzymes was achieved (Fig. S1B). SFK inhibition with dasatinib decreased proliferation of PC-3 cells, consistent with previous findings [11]; knockdown of *IGF-1R* decreased proliferation at 96 hours more than dasatinib did (*P*<0.05; S1C), and the combination of SFK inhibition and *IGF-1R* knockdown further decreased proliferation (*P*<0.05). These findings were confirmed by MTS assay of a 96-hour culture (data not shown). The inhibition of proliferation observed with BMS-754807 was more pronounced in both cell lines than with shIGF-1R, suggesting that the phenotype seen with BMS-754807 is likely due, in part, to the ability of BMS-754807 to inhibit the insulin receptor (IR) as well [26].To specifically examine the effects of BMS-754807 on IGF-1 stimulation of IGF-1R, we determined the effect of increasing doses of the inhibitor on PARP cleavage. As shown in Figure S1D, PARP cleavage was dose-dependent. We also examined the time-dependence of growth inhibition. In the presence of BMS-754807, growth kinetics were inhibited in a time-dependent manner relative to untreated cells (Figure S1E). These results are consistent with BMS-754807 affecting cell growth through increased apoptosis.

In PC-3 and LNCaP cells, treatment with the combination of dasatinib and BMS-754807 increased cell cycle arrest and cell death, relative to either inhibitor alone (Fig. 1B). When examining the specific contribution of each compound, we found that incubation of both cell lines with BMS-754807 increased the proportion of SubG_1_ cells (Fig. 1B), correlating with a higher fraction of annexin-V–positive cells at 48 hours (when cell death was beginning-(Fig. 1C) as well as corresponding with PARP cleavage (Fig. S1F). In contrast, no increases in SubG_1_ fraction and annexin-V–positive cells were observed with the addition of dasatinib only. To screen for possible induction of autophagy, we also tested whether the drugs induced conversion of LC3-I to LC3-II, a process indicative of autophagic activity. As shown in Fig. S1G, neither drug (alone or in combination) produced significant amounts of LC3-II, suggesting autophagy is not a major cause of cell death.

Since only BMS-754807 induced apoptosis, we then performed a similar experiment with shIGF-1R PC-3 cells to determine whether decreased IGF-1R expression specifically increased apoptosis. Indeed, as seen in Fig. 1D, knockdown of *IGF-1R* in PC-3 cells increased the proportion of SubG_1_ as well as annexin-V–positive cells compared with control shRNA cells. Knockdown of *SRC* had little effect (data not shown).

### The combination of dasatinib and BMS-754807 results in more potent inhibition of Akt than does either drug alone

**Figure 2:**
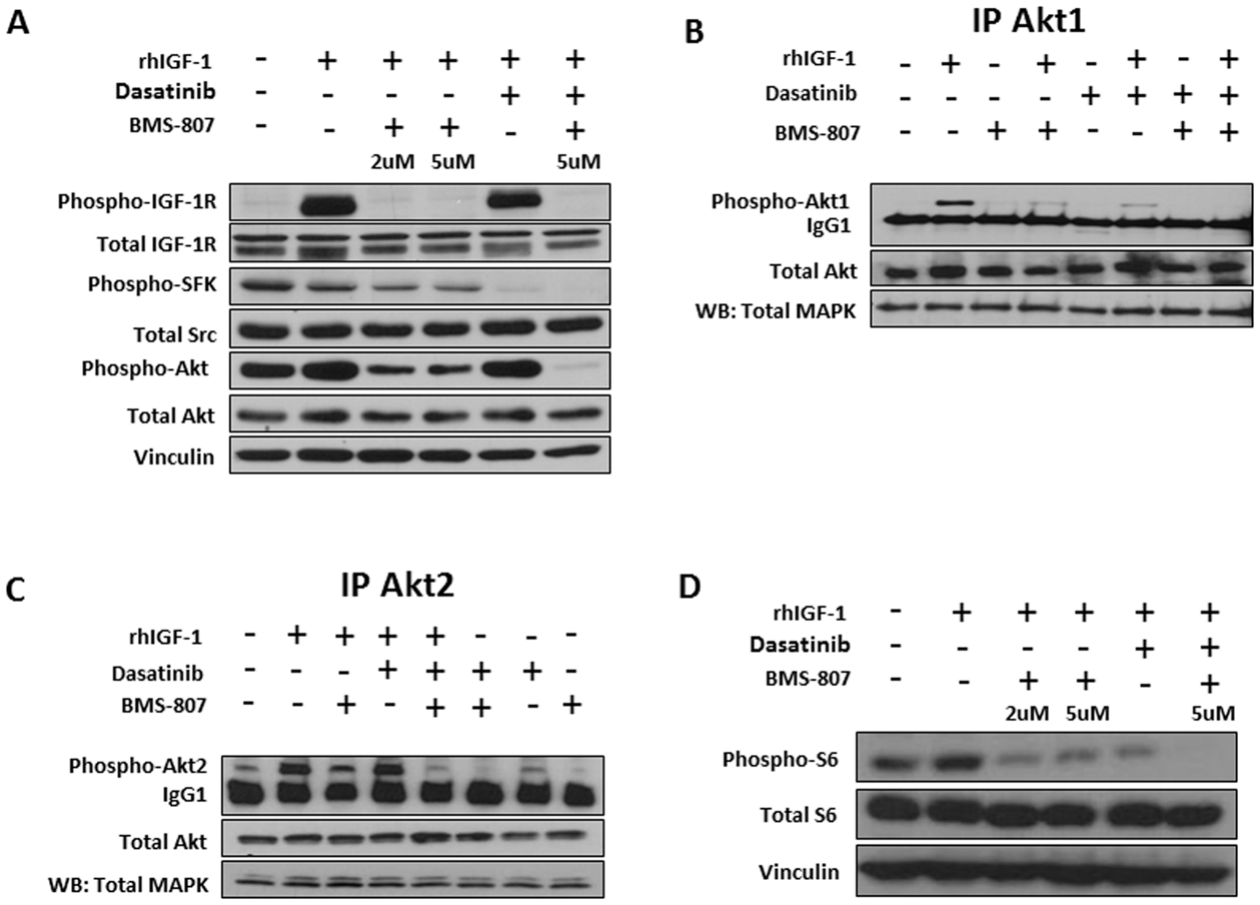
Modulation of IGF-1–induced Akt1 and Akt2 phosphorylation in PC-3 cells by dasatinib (DSA) and BMS-754807 (BMS-807). (A) PC-3 cells were serum starved for 72 hours and then pre-incubated for 2 hours with BMS-754807 at either 2 µM or 5 µM, with dasatinib at 100 nM, or with both agents. After 2 hours, the cells were stimulated with 50 ng/mL recombinant human IGF-1 (rhIGF-1) for 3 minutes. Next, protein was harvested and the (phospho)-proteins IGF-1R, Src, and Akt were determined by western blot (WB). Vinculin was used as the loading control. (B and C) PC-3 cells were stimulated with DSA (100 nM) and BMS-754807 (5 µM) as above, and then the cells were harvested and immunoprecipitated for Akt1 (B) or Akt2 (C), followed by immunoblotting for phospho-Akt. (D) PC-3 cells were treated as in (A), and western blot was run for S6 and phospho-S6.

We next tested whether combined inhibition of SFKs and IGF-1R affected signaling pathways differently than did either agent alone. We first examined potential downstream intermediates of these pathways, Erk1/2 and Akt. Experiments were performed in the presence and absence of IGF-1 because IGF-1 is abundant in the microenvironment of CRPC patients. In serum-starved conditions (to suppress baseline IGF-1R activation), we confirmed target inhibition with both drugs after IGF-1 stimulation (Fig. 2A). Erk1/2 expression or phosphorylation was not affected by either inhibitor alone or when used in combination (Fig. S2). In contrast, Akt activation was partially inhibited by each inhibitor alone and completely inhibited by the combination. To better understand the mechanism of Akt inhibition, we first demonstrated expression of Akt1 and 2 (Fig. 2B, C), but not Akt3 (data not shown), to detectable levels in PC-3 cells. In the absence of IGF-1, Akt2, but not Akt1, is partially activated (Fig. 2B, C). In the presence of IGF-1, Akt1 becomes activated and Akt2 phosphorylation further increases. As shown in Fig. 2B, >80% of Akt1 phosphorylation is inhibited by dasatinib, whereas dasatinib is ineffective in inhibiting IGF-1–induced Akt2 phosphorylation (Fig. 2C). In contrast, BMS-754807 partially, but not completely, reduced both phosphorylation of both Akt1 and 2 in the presence of IGF-1. Dual blockade of IGF-1R and SFK with combined BMS-754807 and dasatinib completely inhibits detectable Akt1 and 2 phosphorylation in the presence of IGF-1 (Fig. 2B, C). To investigate effects on a downstream target of Akt, S6 phosphorylation was examined. In agreement with the results of Akt inhibition, partial inhibition of S6 phosphorylation was seen with both dasatinib and BMS-754807 alone, and complete inhibition was seen with the combination (Fig. 2D), suggesting that inhibiting Akt functions requires both dasatinib and BMS-754807. Thus, the combination of dasatinib and BMS-754807 decreases survival in part, due to complete inhibition of Akt.

### Treatment of primary human xenograft tumors with dasatinib and BMS-754807 is effective *in vivo* and induces tumor apoptosis

**Figure 3:**
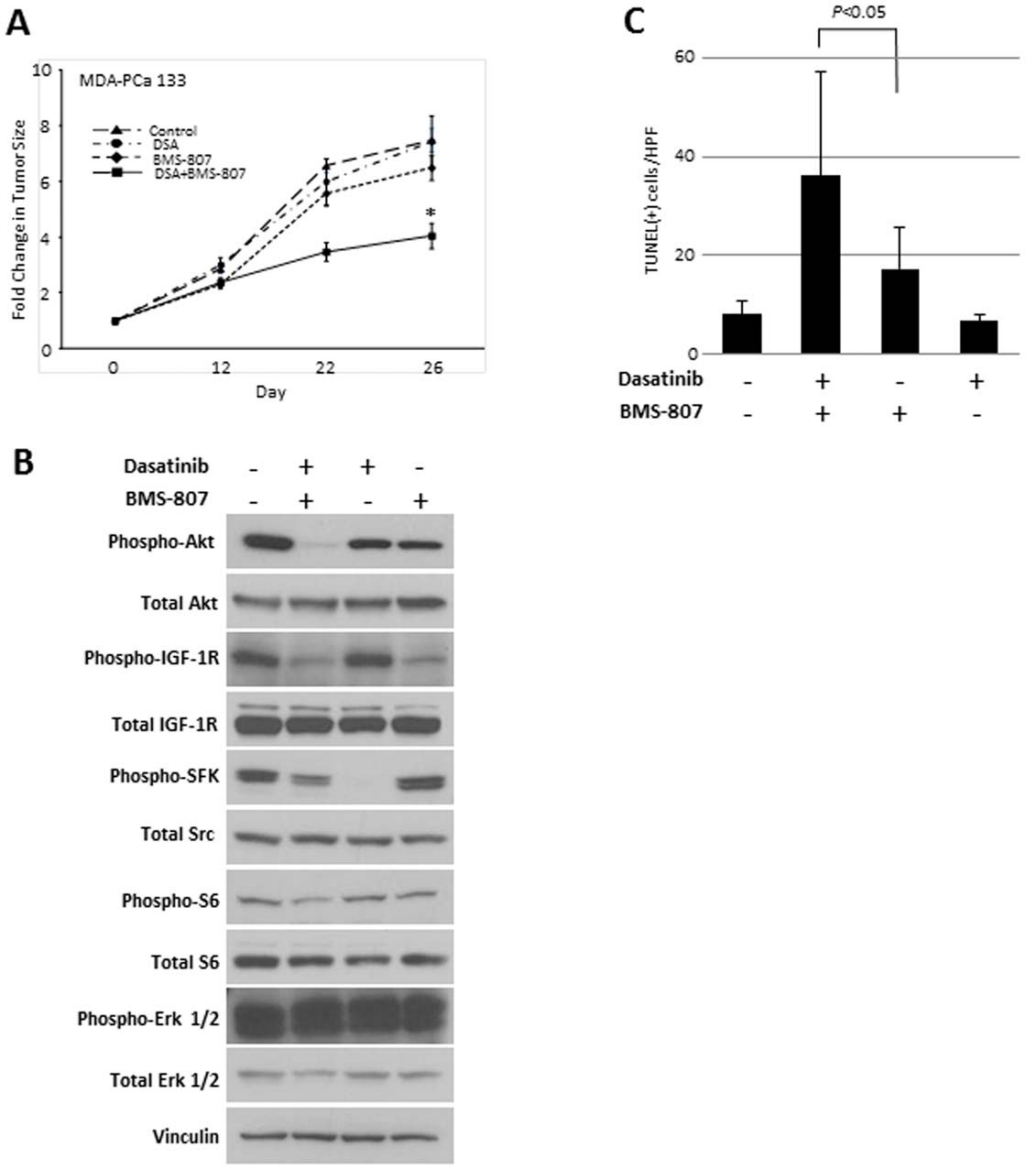
Dasatinib (DSA) and BMS-754807 (BMS-807) inhibit tumor growth after subcutaneous implantation of xenograft MDA PCa 133 cells in nude mice. (A) MDA PCa 133 (derived from bone metastases) cells were implanted subcutaneously in nude mice as described in Methods. Once the tumors reached a volume of 3 mm^3^, the mice were treated with dasatinib and BMS-754807 alone and in combination (n = 4 for each group). Tumors were measured twice a week. Shown is the relative increase in tumor size over time in each group. *P<0.05. (B) The mice were euthanized 16 days after treatment initiation, the tumors were harvested, and western blots were done for the indicated (total and phospho)-proteins. Shown are representative results from a tumor in each treatment group. (C) Quantification of TUNEL staining to detect apoptosis. Shown is the mean(± SEM) number of apoptotic cells per high-power field (HPF) in each group.

To determine the effect of combined inhibition of Src and IGF-1R in a direct xenograft of human PCa cells growing in mouse models, we first treated mice bearing the primary human xenograft MDA PCa 133 tumors (castrate-resistant, AR positive) with dasatinib and BMS-754807 alone and in combination(n = 4 for each group). As shown in Fig. 3A, at 3 weeks, there was an increase in tumor size in the control group as well as the single-drug groups, whereas the tumor growth rate was significantly reduced in the combination group. This effect was even more pronounced on day 26, at which time the mice were euthanized.

Immunoblots on flash-frozen tumors harvested from the single agent–treated mice demonstrated inhibition of Src phosphorylation by dasatinib and IGF-1R phosphorylation by BMS-754807, demonstrating that these drugs hit their principal targets. Exactly as we observed in the *in vitro* studies, Akt and S6 phosphorylation was only partially inhibited by each drug alone. In contrast, robust inhibition of Akt and S6 phosphorylation was observed in the combination group (Fig. 3B). Further, apoptosis was increased, as determined by TUNEL staining of the BMS-754807–treated tumors and further increased in the combination group (*P* = 0.01; Fig. 3C). These results suggest that combined inhibition of Src and IGF-1R is effective in primary human xenografts, mediated in part by near-complete inhibition of Akt phosphorylation.

To examine orthotopic tumor inhibition, PC-3 LG cells (chosen for their androgen receptor [AR]-negative status and robust growth in orthotopic models) were injected into the prostates of nude mice and treated as described in the Methods section. Four weeks after cell injection, the mice were killed and the tumors excised and weighed (Fig. S3A). Representative tumors from the 4 treatment groups (total n = 8 for each group) are shown in Fig. S3B. There was no significant difference in tumor weight between the control, dasatinib, and BMS-754807 groups at the concentrations used (as expected), but the mice treated with the combination of drugs had significantly smaller tumors (*P*<0.03; Fig. S3 A,B).

### Dasatinib and BMS-754807 inhibit PC3-MM2–induced bone destruction and decrease bone turnover markers *in vivo*

**Figure 4:**
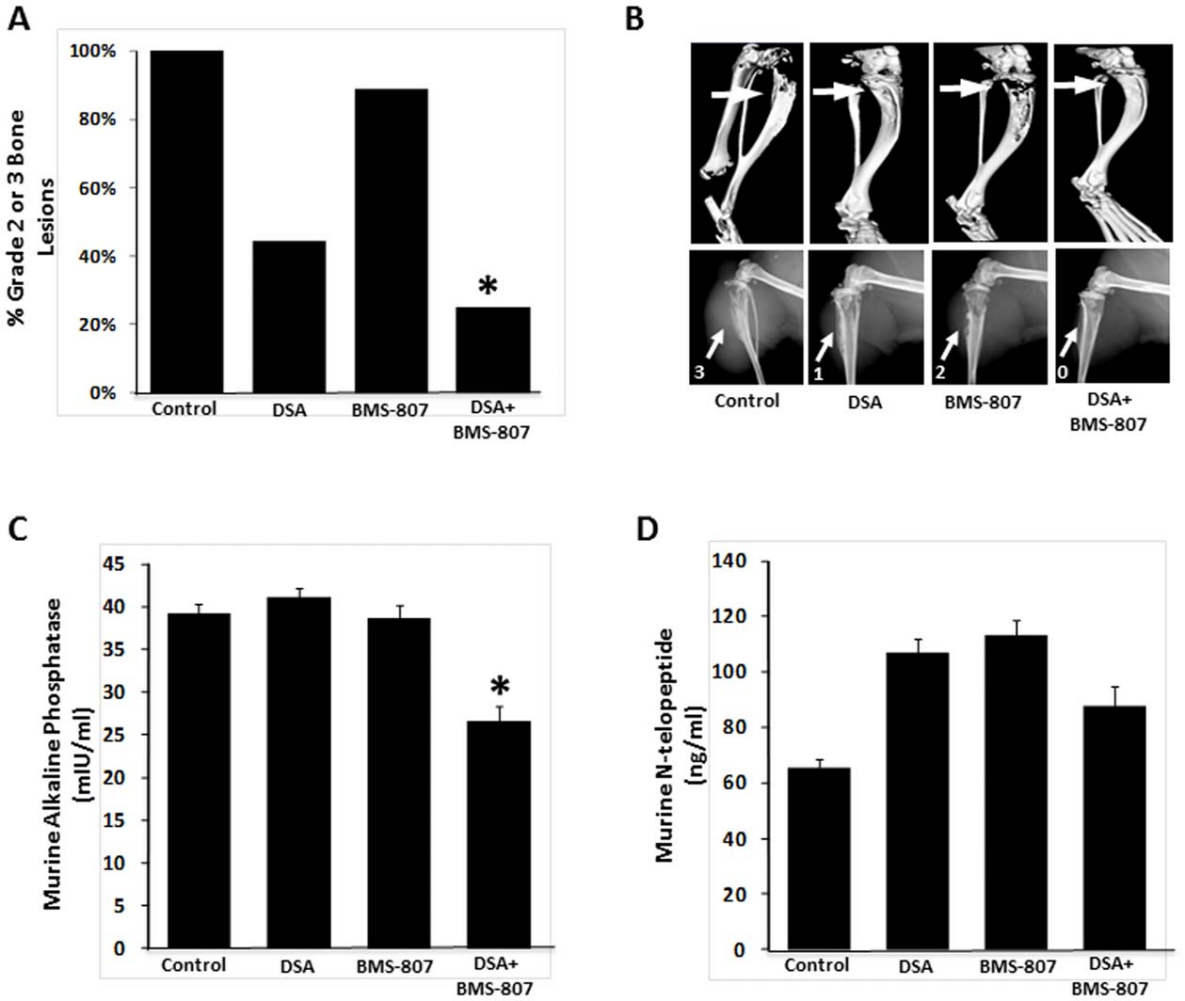
Dasatinib (DSA) and BMS-754807 (BMS-807) decrease intratibial bone destruction and tumor formation and modulate bone turnover markers. Bone-destructive PC3-MM2 cells were injected intratibially in nude mice. Following treatment with dasatinib, BMS-754807 alone and combined, the mice were euthanized, and x-rays and computed tomographic (CT) scanning of the long bones were done (control, n = 10; dasatinib, n = 9; BMS-754807, n = 9; combination, n = 8). Blood was collected for serum bone turnover markers. (A) The degree of bone destruction was blindly graded in a semiquantitative fashion based on x-rays from all mice. The graph displays the proportion of high-grade lesions (i.e., 2 or 3) in each treatment group. * *P*<0.05 combination vs. dasatinib only. (B) Representative CT scans (top) and x-rays (bottom) from the mice in each group. Arrows and numbers indicate the lesion site and the grade of bone destruction. (C and D) Serum levels of murine alkaline phospatase (C) and N-telopeptide (D) were determined by ELISA. Bars indicates SEM. * *P*<0.05.

To investigate whether dasatinib and BMS-754807 reduced PCa-induced bone turnover, we injected PC3-MM2 cells intratibially into nude mice. Two weeks after injection, the mice were treated for 4 weeks with either drug alone or in combination (control, n = 10; dasatinib, n = 9; BMS-754807, n = 9; combination, n = 8). At this time, X-rays were taken and mice were exsanguinated following euthanization. The involved bones were harvested for computed tomography. PCa cell–induced tumor growth and bone destruction, as measured on plain x-rays, was scored in blinded fashion as described in Methods. Whereas, as expected, treatment with dasatinib alone exhibited some inhibition of bone destruction, the combination of dasatinib and BMS-754807 more effectively inhibited intratibial tumor growth in the bone (Fig. 4A, B). However, only the combination of drugs significantly reduced the serum levels of bone turnover markers (Fig. 4C, D), indicating modulation of both the PCa cells and their bone microenvironment, thus interfering with the vicious cycle [10]. Whereas the modification of murine N-telopeptide, a surrogate for osteoclast activity, is indicative of inhibition of the osteolytic PC3-MM2 cell line, the observed decrease in murine alkaline phosphatase (representing osteoblast activity) highlights the interdependence of these cells in the process of bone turnover. Dasatinib alone has been shown to induce osteoblast maturation [38]; thus possibly increasing RANKL secretion, which in turn normally activates osteoclasts. However, as shown in the Src-/- mouse [39], Src is also an important regulator of osteoclast function, and this is confirmed by bone preservation in dasatinib-alone treated cells (Fig. 4B). However, in the models used, dasatinib as a single agent at the lower concentration used is insufficient to inhibit tumor growth, suggesting that under these conditions, expression of RANKL at least partially activates osteoclast function, but that the addition of BMS-754807 to dasatinib likely inhibits osteoblast maturation, further decreasing osteoclast activity. This result appears to occur in each model, and thus is a consistent feature of this drug combination.

## Discussion

Emerging evidence suggests that successful treatment for advanced PCa will require targeting both the tumor and the microenvironment [10]. However, multiple bidirectional signaling occurs between tumor and bone, and these signals regulate redundant and overlapping pathways that contribute to tumor growth at the metastatic site. Thus, cytokines abundant in metastatic PCa to the bone may play a critical role in activating Akt in tumor cells that cannot be overcome by use of some TKI, such as dasatinib. These results suggest that combinations of inhibitors will be required to improve patient survival. Examples of tumor/microenvironment interactions are the importance of Src family members activated in PCa [40], [41] in tumor growth and progression, as well as the importance of Src in osteoclast function [39]; thus, Src targeting affects both the tumor and microenvironment. This strategy has led to a subset of patients who experience durable responses in a phase 1/2 dasatinib plus docetaxel trial [12], leading to a large randomized phase 3 trial with docetaxel in men with advanced metastatic PCa (NCT00744497). We have been assessing pathways whose inhibition might complement Src inhibitors. We have focused on the IGF-1/IGF-1R pathway because the activation of this pathway is important in PCa, the abundant expression of IGF-1 [42] in osteoblasts leads to frequent activation of IGF-1R in tumor cells [43], [44] and because IGF-1R may signal through compensatory pathways that blunt the effectiveness of Src inhibitors such as dasatinib. [45]. Therefore, we sought to test whether inhibiting signaling axes in PCa mediated by Src and IGF-1R would cooperatively inhibit tumorigenic functions of PCa cells.

In this study, we utilized these inhibitors together with genetic approaches to modulate the Src and IGF-1R/IR pathways to examine effects on proliferation, apoptosis, and cell signaling. We demonstrated that silencing of *IGF-1R* affected proliferation and cell survival, in agreement with previous findings using monoclonal antibodies against IGF-1R [22], and was significantly enhanced with the addition of the SFK inhibitor dasatinib. Similarly to shRNA-mediated knockdown of *IGF-1R*, BMS-754807 affected proliferation and increased apoptosis, whereas shRNA to *SRC* had less effect on proliferation, as demonstrated previously [11]. These results suggest that Src and IGF-1R regulate overlapping pathways and their combined inhibition may be more effective than inhibiting either kinase alone in treating PCa cells, especially when IGF-1 is abundant, such as in bone metastases. Indeed, we demonstrated enhancing effects when combining BMS-754807 with dasatinib with respect to growth inhibition and apoptosis. Since dasatinib alone did not produce a notable phenotype in our models, its combination with BMS-754807 by definition enhances its effects rather than showing synergy [46].

Our *in vitro* observations were substantiated in several *in vivo* models of PCa growth, including an AR-positive castrate-resistant primary human xenografts (MDA PCa 133) that cannot be grown in tissue culture and orthotopic and bone tumors derived from PC-3 cells (AR negative). In every model, the combination of the two drugs more significantly inhibited tumor growth than did either alone. This is likely a combination of apoptosis induced by BMS-754807, and decreased proliferation/invasion by dasatinib, which all lead to an enhanced tumor control in vivo despite the modest combinatorial effects in vitro.

The modulation of the bone-tumor interaction was also reflected in the observed changes of serum bone-turnover markers *in vivo*. Interestingly, while the combination of dasatinib and BMS-754807 normalized levels of N-telopeptide (**Fig. 4D**), a marker for osteoclast activity, this result was not observed in mice treated with dasatinib only. Dasatinib has been shown to be an inhibitor of osteoclast function *in vitro* [47] and *in vivo* [48] and in our studies, dasatinib alone did preserve bone structure (Fig. 4B). Thus, a decrease in N-telopeptide with dasatinib might have been expected. However, our *in vivo* model in which an aggressive osteolytic cell line, PC3-MM2 was injected intratibially, and mice were dosed with a concentration of dasatinib that alone did not induce significant tumor inhibition, suggests additional interactions between the tumor and its host are critical for tumor growth, despite effects of dasatinib on osteoclast function. More importantly, the study by Vandyke et al. [48] examined the *in vivo* effects by treating mice with dasatinib for as many as 12 weeks. Although no tumor cells were injected to the bone, similar to our observations, at 4 weeks of treatment there was a modest increase in the serum osteoclast marker CTX-1 (C-terminal collagen cross-links), and a decrease of CTX-1 by dasatinib was seen only after week 8 of treatment. Hence, our observations of a slight increase in N-telopeptide after four weeks of treatment agree with this study. Clearly, more research is necessary to elucidate the complex relationships between dasatinib effects on osteoblasts, osteoclasts, and tumor growth.

To better understand the molecular basis of the enhanced effects of BMS-754807 and dasatinib, we examined downstream pathways potentially regulated by Src and IGF-1R. While Erk1/2 phosphorylation was unaffected, nearly complete inhibition of Akt phosphorylation was observed with the combination of dasatinib and BMS-754807.These findings are in keeping with recent data showing in breast cancer that Erk1/2 phophorylation is not mediated by IGF-1, but rather insulin and IGF-2 [49]. Our findings are supported by two recent reports that identified activated Akt as a downstream effector of IGF-1–mediated suppression of apoptosis in PC-3 cells [23] and also showed that *in vitro* and *in vivo* inhibition of migration/invasion by the Src inhibitor bosutinib is accompanied by inhibition of Akt phosphorylation [50]. Wu et al recently showed Src inhibition affects Akt phosphorylation and induces autophagy in PC3 cells [51]. However, we have observed residual Akt phosphorylation independent of Src and IGF-1R pathways (Fig. 2C), which likely is explained by other kinases increased during prostate cancer progression, such as c-Met [52] and Axl [53]. Additionally, Akt is activated downstream of integrin activation. Knockdown of β1 integrin in PC3-MM2 cells leads to decreased Akt phosphorylation (Jin and Gallick, unpublished data). As each of these pathways activate Src, dasatinib appears to have some effect in inhibiting at least Akt1 from divergent mediators of PCa progression.

**Figure 5:**
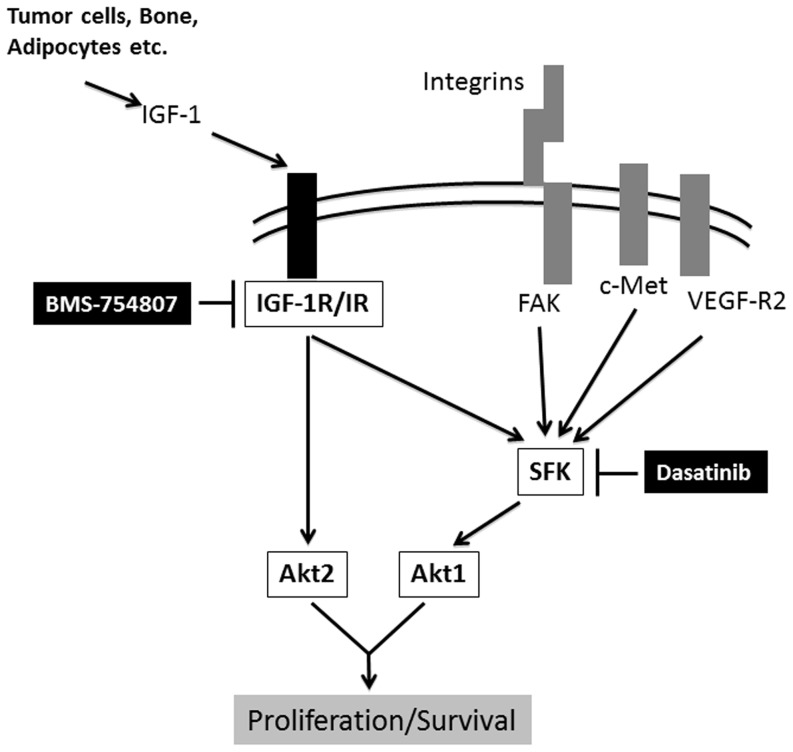
Dasatinib and BMS-754807 have different effects on tumorigenic properties of PCa cells. Circulating IGF-1 from multiple sources (bone, tumor cells, adipocytes, etc.) binds to IGF-1R, which leads to downstream activation of Akt and suppression of apoptosis. BMS-754807 inhibits IGF-1R phosphorylation and thus leads to partial inhibition of Akt1 and Akt2. While Akt2 phosphorylation is Src independent, Akt1 phosphorylation is also Src mediated, so full blockade is dependent on dual inhibition of Src and IGF-1R. Activation of IGF-1R, but also of many other cell surface receptors (integrins, vascular endothelial growth factor-receptor [VEGF-R], Axl, c-Met, etc.), involves Src activation, which increases PCa cell motility, migration, and invasion. Dasatinib inhibits Src phosphorylation, thereby decreasing invasive/migratory properties of PCa cells. Since Akt is activated by both IGF-1R and Src, blockade of either pathway alone will only partially inhibit Akt phosphorylation, whereas the dual blockade almost completely abrogates Akt phosphorylation. FAK: focal adhesion kinase.

We therefore hypothesize that independent activation of PI3K by Src (which directly binds PI3K) and IGF-1R (activation of which leads to PI3K association with IRS and activation [45]) leads to the inability of dasatinib or BMS-754807 to completely inhibit Akt functions if used as single agents. Our results further suggest that constitutive Akt2 phosphorylation overcomes effects of inhibition of Src family kinases alone by dasatinib (Fig. 5). Support for this hypothesis comes from recent work of Paccez et al., who demonstrated that downregulation of Akt by Axl knockdown could be overcome by addition of IGF-1 [53], although whether Akt2 phosphorylation was specifically responsible for overcoming Axl resistance was not examined.

The differential effects of Src inhibition on Akt1 and Akt2 phosphorylation are currently under investigation. Overall, our results suggest that strategies to interfere with the Src and IGF-1R pathways in PCa are effective and that using small tyrosine-kinase inhibitors (TKIs) against both targets is more potent than specific targeting, likely reflecting inhibition of related targets by the TKIs. Thus, this combination is promising for future clinical trials in advanced PCa. BMS-754807 is currently being tested in several phase 2 trials in combination with letrozole (NCT01225172) and trastuzumab (NCT00788333) in breast cancer and with cetuximab in multiple tumor types (NCT00908024). The safety and tolerability were established in a recent dose-escalation phase 1 trial of BMS-754807 (NCT00569036), in which the maximal tolerated dose had not yet been reached at the time of the study's presentation [54]. Dasatinib was also combined with docetaxel in a phase 3 randomized trial in ∼1500 men with CRPC (NCT00744497). Accrual of patients for that study is finished and overall survival results are pending [6]. Therefore, our study is of potential clinical importance as both inhibitors used in this work (although not in combination) are currently in clinical trials, and this work provides strong rationale for combining these two agents in men with CRPC.

## Supporting Information

Figure S1 (A) Expression of SFK proteins Src, Yes, Fyn, and Lyn was demonstrated by Western Blot in AR-negative PC-3 cells, AR-positive LNCaP cells, and in the primary human AR-expressing CRPC xenograft MDA PCa 133. (B) Knockdown of Src and IGF-1R in PC-3 and LNCaP cells. (C) PC-3–shIGF-1R or control cells were incubated for up to 72 hours with and without dasatinib (DSA; 100 nM), and cell numbers were determined as described in Methods. (D) Dose-dependent PARP cleavage in PC-3 cells induced by BMS-754807. After serum deprivation, PC-3 cells were incubated for 48 hours in presence of IGF-1 with increasing concentrations of BMS-754807, and cleaved PARP was determined by western blot as described in Methods. Vinculin served as loading control. Bars, from one representative experiment, represent normalization of cPARP to loading control. (E) Time dependence of BMS-754807 effects. PC-3 cells (3×10^4^) were plated in triplicate with or without 1 µM BMS-754807. Cell were counted daily up to 96 hours as described in Methods. (F) PC-3 cells were incubated with 5 µM BMS-754807, 100 nM DSA, and both for 48 hours. Protein was extracted from the cells, and western blot for cleaved PARP was performed as described in Methods. Vinculin serves as loading control. (G) Western blot for LC3 in PC3 and LNCaP after treatment as decribed in (F). Conversion of LC3-I (18 kDa) to LC3-II (16 kDa; see arrow) is indicative of autophagocytic activity. GAPDH served as loading control.
(TIF)

Figure S2 **Dasatinib and BMS-754807 (BMS-807) do not modulate Erk1/2 phosphorylation.** PC-3 cells were serum starved for 72 hours and then pre-incubated for 2 hours with BMS-754807 at either 2 µM or 5 µM, with dasatinib at 100 nM, or with both. After 2 hours, the cells were stimulated with 50 ng/mL rhIGF-1 for 3 minutes. Then protein was harvested and the (phospho)-proteins Erk1/2 were determined by western blot. Vinculin was used as the loading control.
(TIF)

Figure S3 **Dasatinib (DSA) and BMS-754807 (BMS-807) inhibit tumor growth after orthotopic injection of PC3-LG cells into the prostates of nude mice.** The mice (n = 8 per group) were treated with dasatinib and BMS-754807 alone and in combination at the doses described in Methods starting 10 days after injection of the tumor cells. (A) The prostates were removed 4 weeks after injection and the tumor weight was measured. Horizontal lines depict the median (± SD) tumor weight for each group. n.s., not statistically significant. (B) Representative photographs of tumors harvested from each group.
(TIF)
