# Supplementary figures and images for: Combined Inhibition of IGF-1R/IR and Src Family Kinases Enhances Antitumor Effects in Prostate Cancer by Decreasing Activated Survival Pathways
Source: PLoS One. 2012 Dec 26;7(12):e51189. doi: 10.1371/journal.pone.0051189 (PMC3530555; doi:10.1371/journal.pone.0051189)

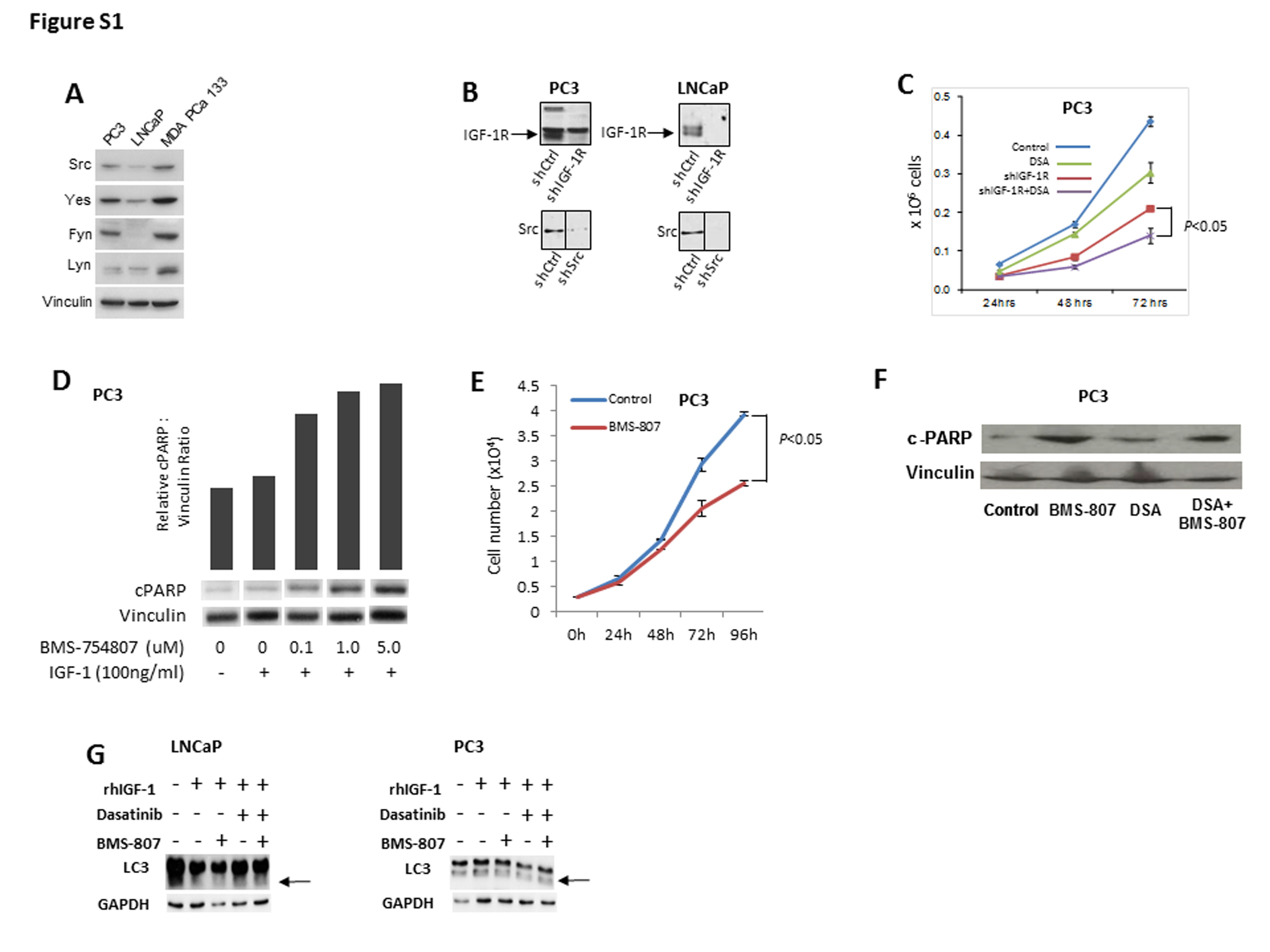

Supplement: Figure S1 — (A) Expression of SFK proteins Src, Yes, Fyn, and Lyn was demonstrated by Western Blot in AR-negative PC-3 cells, AR-positive LNCaP cells, and in the primary human AR-expressing CRPC xenograft MDA PCa 133. (B) Knockdown of Src and IGF-1R in PC-3 and LNCaP cells. (C) PC-3–shIGF-1R or control cells were incubated for up to 72 hours with and without dasatinib (DSA; 100 nM), and cell numbers were determined as described in Methods. (D) Dose-dependent PARP cleavage in PC-3 cells induced by BMS-754807. After serum deprivation, PC-3 cells were incubated for 48 hours in presence of IGF-1 with increasing concentrations of BMS-754807, and cleaved PARP was determined by western blot as described in Methods. Vinculin served as loading control. Bars, from one representative experiment, represent normalization of cPARP to loading control. (E) Time dependence of BMS-754807 effects. PC-3 cells (3×10^4^) were plated in triplicate with or without 1 µM BMS-754807. Cell were counted daily up to 96 hours as described in Methods. (F) PC-3 cells were incubated with 5 µM BMS-754807, 100 nM DSA, and both for 48 hours. Protein was extracted from the cells, and western blot for cleaved PARP was performed as described in Methods. Vinculin serves as loading control. (G) Western blot for LC3 in PC3 and LNCaP after treatment as decribed in (F). Conversion of LC3-I (18 kDa) to LC3-II (16 kDa; see arrow) is indicative of autophagocytic activity. GAPDH served as loading control. (TIF) [file pone.0051189.s001.tif]

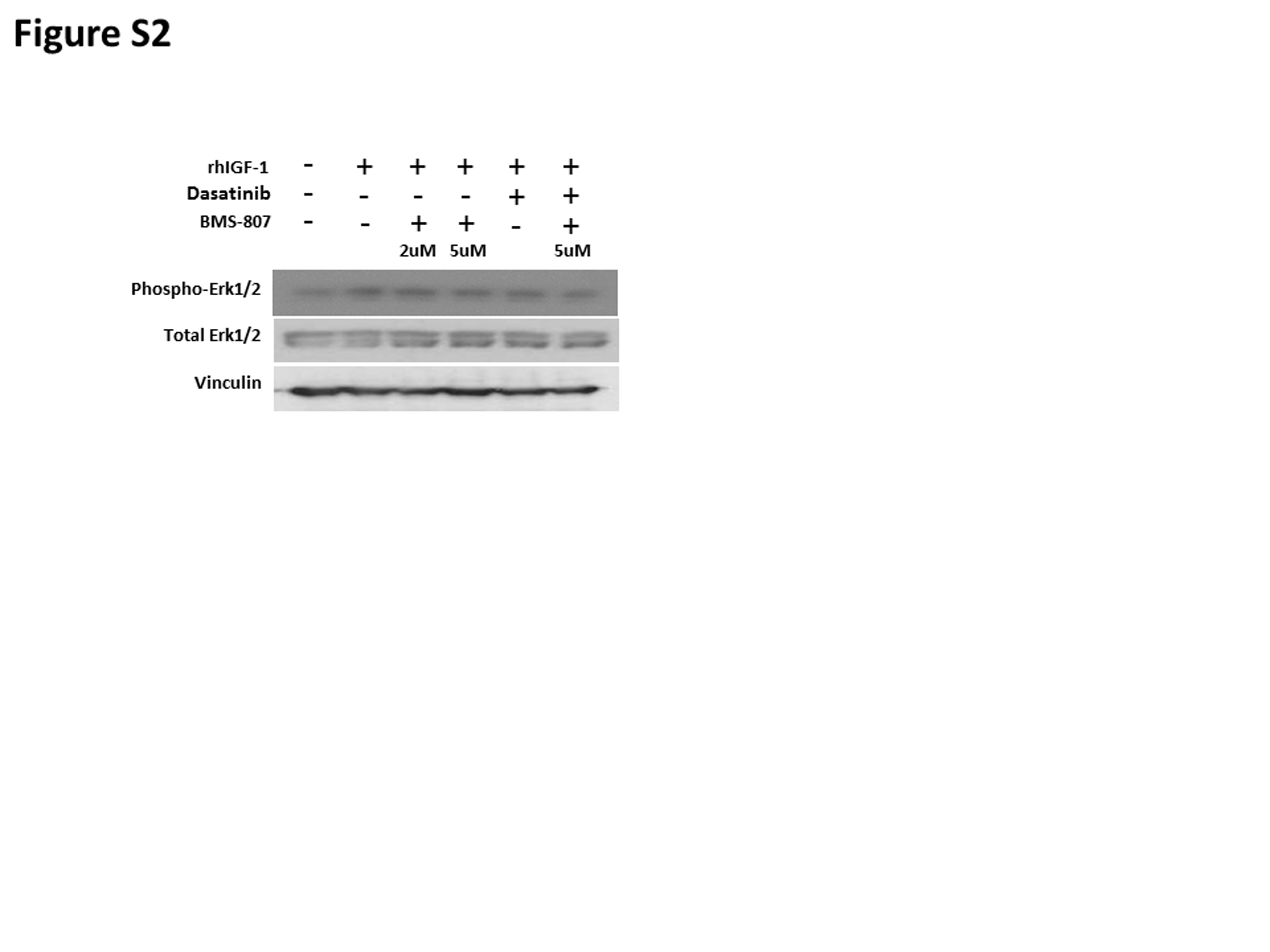

Supplement: Figure S2 — Dasatinib and BMS-754807 (BMS-807) do not modulate Erk1/2 phosphorylation. PC-3 cells were serum starved for 72 hours and then pre-incubated for 2 hours with BMS-754807 at either 2 µM or 5 µM, with dasatinib at 100 nM, or with both. After 2 hours, the cells were stimulated with 50 ng/mL rhIGF-1 for 3 minutes. Then protein was harvested and the (phospho)-proteins Erk1/2 were determined by western blot. Vinculin was used as the loading control. (TIF) [file pone.0051189.s002.tif]

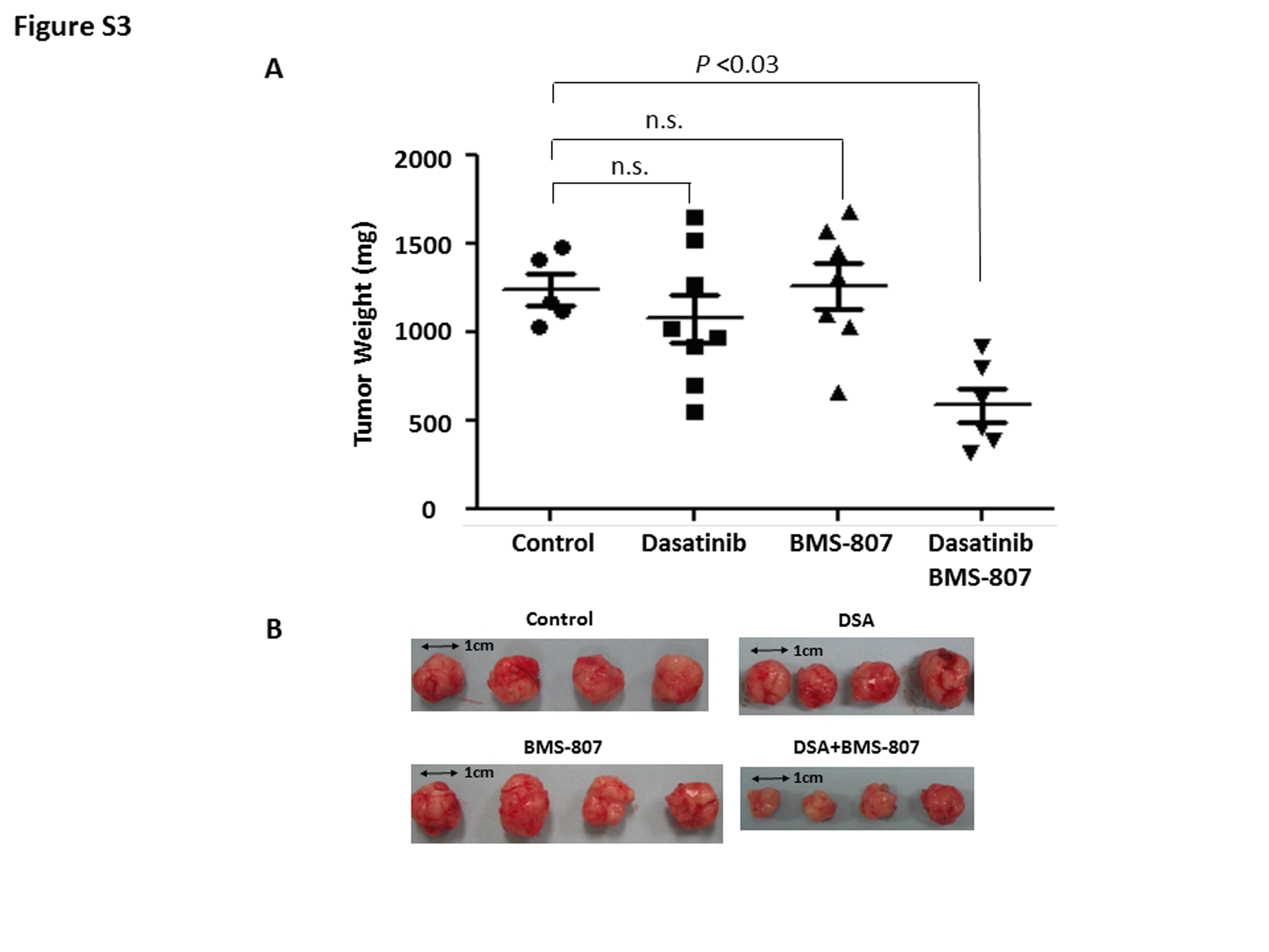

Supplement: Figure S3 — Dasatinib (DSA) and BMS-754807 (BMS-807) inhibit tumor growth after orthotopic injection of PC3-LG cells into the prostates of nude mice. The mice (n = 8 per group) were treated with dasatinib and BMS-754807 alone and in combination at the doses described in Methods starting 10 days after injection of the tumor cells. (A) The prostates were removed 4 weeks after injection and the tumor weight was measured. Horizontal lines depict the median (± SD) tumor weight for each group. n.s., not statistically significant. (B) Representative photographs of tumors harvested from each group. (TIF) [file pone.0051189.s003.tif]
